# RNA Interference-Guided Targeting of Hepatitis C Virus Replication with Antisense Locked Nucleic Acid-Based Oligonucleotides Containing 8-oxo-dG Modifications

**DOI:** 10.1371/journal.pone.0128686

**Published:** 2015-06-03

**Authors:** Margit Mutso, Andrei Nikonov, Arno Pihlak, Eva Žusinaite, Liane Viru, Anastasia Selyutina, Tõnu Reintamm, Merike Kelve, Mart Saarma, Mati Karelson, Andres Merits

**Affiliations:** 1 Institute of Technology, University of Tartu, Tartu, Estonia; 2 GeneCode, Ltd., Tallinn, Estonia; 3 Department of Gene Technology, Tallinn University of Technology, Tallinn, Estonia; 4 Institute of Biotechnology, University of Helsinki, Helsinki, Finland; 5 Institute of Chemistry, University of Tartu, Tartu, Estonia; International Centre for Genetic Engineering and Biotechnology, ITALY

## Abstract

The inhibitory potency of an antisense oligonucleotide depends critically on its design and the accessibility of its target site. Here, we used an RNA interference-guided approach to select antisense oligonucleotide target sites in the coding region of the highly structured hepatitis C virus (HCV) RNA genome. We modified the conventional design of an antisense oligonucleotide containing locked nucleic acid (LNA) residues at its termini (LNA/DNA gapmer) by inserting 8-oxo-2’-deoxyguanosine (8-oxo-dG) residues into the central DNA region. Obtained compounds, designed with the aim to analyze the effects of 8-oxo-dG modifications on the antisense oligonucleotides, displayed a unique set of properties. Compared to conventional LNA/DNA gapmers, the melting temperatures of the duplexes formed by modified LNA/DNA gapmers and DNA or RNA targets were reduced by approximately 1.6-3.3°C per modification. Comparative transfection studies showed that small interfering RNA was the most potent HCV RNA replication inhibitor (effective concentration 50 (EC_50_): 0.13 nM), whereas isosequential standard and modified LNA/DNA gapmers were approximately 50-fold less efficient (EC_50_: 5.5 and 7.1 nM, respectively). However, the presence of 8-oxo-dG residues led to a more complete suppression of HCV replication in transfected cells. These modifications did not affect the efficiency of RNase H cleavage of antisense oligonucleotide:RNA duplexes but did alter specificity, triggering the appearance of multiple cleavage products. Moreover, the incorporation of 8-oxo-dG residues increased the stability of antisense oligonucleotides of different configurations in human serum.

## Introduction

The application of complementary DNA or RNA molecules or their derivatives for the modulation biological functions of specific RNA(s) is referred to as antisense technology. Antisense oligonucleotides (ASOs) are the major class of antisense agents used for sequence-specific RNA knockdown [1], and they can also be used to modulate RNA synthesis, maturation and transport. Two different mechanisms account for the inhibitory properties of ASOs. The first mechanism is typically mediated by the steric inhibition of translation machinery operating on the targeted RNA. In general, this mechanism is not associated with the destruction of targeted molecules, and, accordingly, it is most effective for coding RNAs if the ASO target site overlaps with or is located upstream of the initiation codon [2]. The second mechanism relies on the ability of ribonuclease H (RNase H), a ubiquitous group of cellular enzymes, to cleave the RNA part of the heteroduplexes formed between DNA ASOs and targeted RNA [3,4]. This mechanism results in the degradation of the targeted RNA and is therefore effective regardless of the position of the ASO binding site [2].

The activity of ASOs depends on many factors, including the efficiency of cell entry, the stability of the complex formed with the targeted RNA and the resistance of the ASO to enzymatic degradation. The low potency of standard RNA and DNA ASOs results from their poor uptake and extremely short intracellular and serum half-lives. Sugar moiety and phosphate backbone modifications have been used to increase the resistance of ASOs to degradation. Some of these modifications also increase the binding efficiency of ASOs to their target sequences [5] and/or may be beneficial for cell entry. However, only phosphorothioate-s [6], boranophosphate- [7], oxepane- [8], cyclohexene- [9], and fluoro-arabino (FANA)-modified ASOs [10] have been reported to activate RNase H upon binding to targeted mRNA. In contrast, fully modified N3’,P5’-phosphoramidates [11], morpholinos [12], peptide nucleic acids (PNA) [13], tricyclo-DNA [14], 2’-O-methyl locked nucleic acids (LNA) and 2’-O-methoxyethyl RNAs [15] lack this property. To overcome this issue, co-polymers of 2’-O-methyl RNA [16], FANA [17], PNA or LNA [18–20] with DNA have been developed. ASOs containing LNA residues at their termini (hereafter, ASOs with several terminal LNA monomers and internal DNA residues are termed LNA/DNA gapmers) are more effective activators of RNase H-mediated cleavage than 2’-O-methyl RNA/DNA gapmers or all-DNA ASOs [19].

The nucleobase moiety represents an alternative option for ASO modification. Several heterocyclic base modifications in ASOs have been described (reviewed in [21]). However, only a few of those modifications have been analyzed for their ability to activate RNase H. Thus far, ASOs with modified nucleobases (such as 5-(N-aminohexyl)carbamoyl-2’-dU [22] and G-clamps [23]) have been found to be worse RNase H activators than non-modified DNA oligonucleotides. The majority of sugar moiety, phosphate backbone, and nucleobase modifications increase the melting temperature (Tm) of ASO duplexes with DNA and RNA [24,25]. Furthermore, ASOs containing both LNA bases and phosphorothioate modifications possess excellent serum stability and long *in vivo* half-lives, enabling their successful use in clinical trials [26].

**Fig 1:**
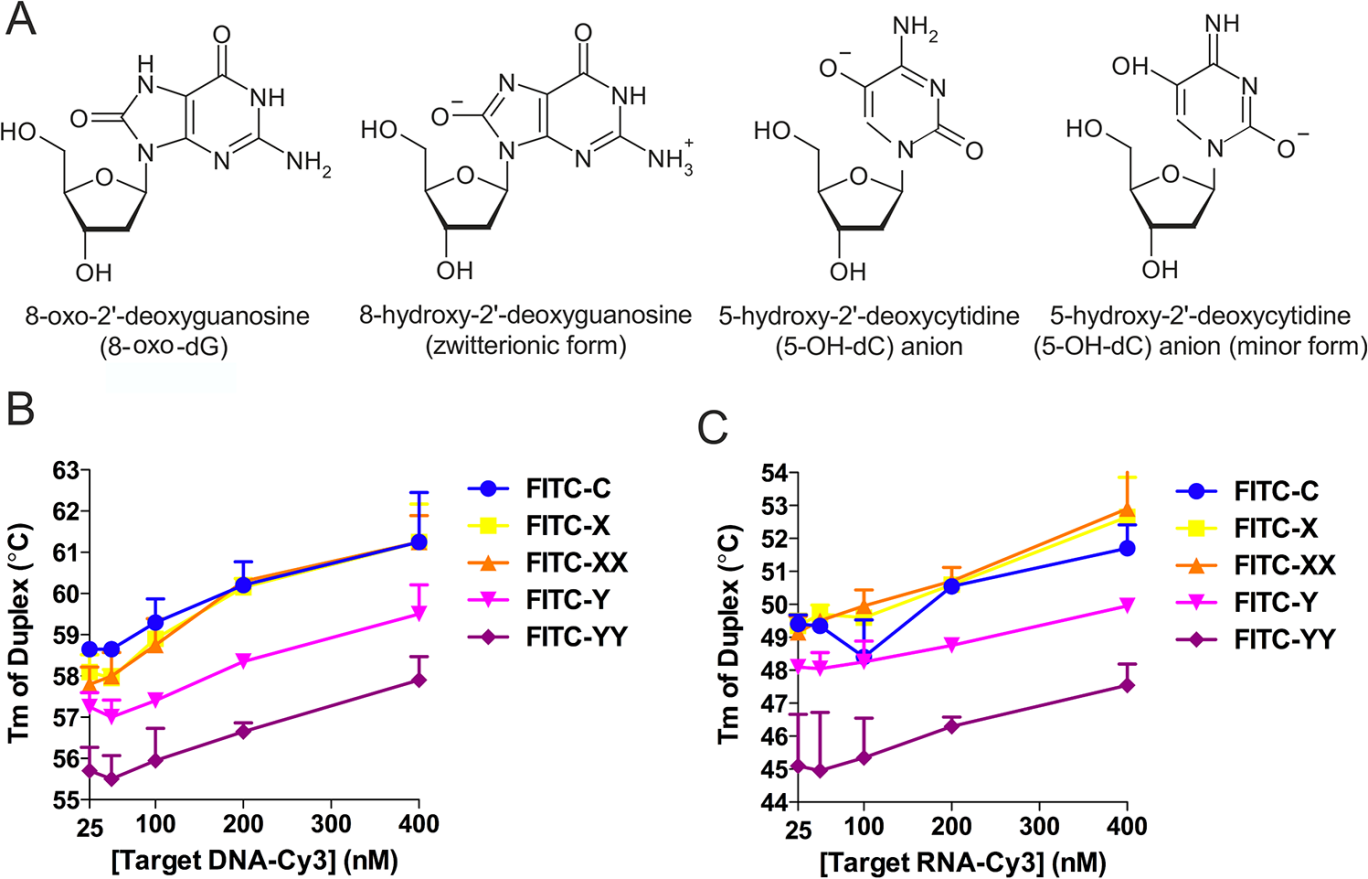
Incorporation of 8-oxo-dG, but not 5-OH-dC, reduces the Tm of ASO:DNA and ASO:RNA duplexes. **(A)** Structures of 8-oxo-dG, its zwitterionic (minor) form and 5-OH-dC (common and minor forms). **(B, C)** The effects of modified nucleobases on the Tm of ASO:DNA **(B)** and ASO:RNA **(C)** duplexes as measured by FRET. Target DNA or RNA oligonucleotides (Table 2) were labeled with Cy3 at the 3’-end, and modified oligonucleotide probes and controls were labeled with FITC at the 5’-end. Probe:target hybridization was quantified by measuring the decrease in FITC fluorescence due to the energy transfer to the Cy3 fluorochrome attached to the hybridized target. Increasing target concentrations (25, 50, 100, 200, and 400 nM) were used (*x*-axis), whereas the probe concentration remained constant at 50 nM in all experiments. The experimentally measured Tm is presented on the *y*-axis. Y (X) and YY (XX) probes contained one or two 8-oxo-dG (5-OH-dC) modifications, respectively. C, unmodified control probe. The error bars represent the standard deviation of three independent experiments.

The 8-oxo-2’-deoxyguanosine (8-oxo-dG) residue contains a minimally modified nucleobase, which is naturally occurring and can result from oxidative DNA damage. In the context of ASO, 8-oxo-guanine forms 3- to 4-fold weaker bonds with complementary cytosine (compared to non-modified guanine) [27], which results in a decrease in the Tm of the ASO:DNA duplexes [28–30]. However, both 8-oxo-dG [31] and 5-hydroxy-2’-deoxycytidine (5-OH-dC) [32], another product of DNA oxidization, have not only major but also minor zwitterionic and ionic tautomeric isomers, respectively (Fig 1A). Interestingly, theoretical quantum chemical calculations performed on the minor tautomeric form of 8-oxo-dG and anion of 5-OH-dC suggest that they exhibit abnormally strong bonding with their respective normal nucleobases in an aqueous environment [33]. It is not known how these effects might contribute to the efficiency of ASOs containing such modified residues.

The *in vivo* delivery of therapeutic nucleic acids to target tissues and organs represents another important problem that has been only partly solved (reviewed [34]). In the absence of specific delivery vehicles, the main target of ASOs in both rodent [35] and non-human primate [36,37] models is liver. Targeting other organs, including kidney [35,37], heart, diaphragm, lung, fat, gastrocnemius and quadriceps, is less efficient [35]. Clinical trials have shown that miravirsen, an experimental ASO drug that targets the microRNA miR122, spontaneously enters the liver [26].

As ASOs have a clear potential for the treatment of liver-associated pathologies, we chose hepatitis C virus (HCV) RNA as a target for modified ASOs. HCV is a major human pathogen that affects the lives of over one hundred million people. Its positive polarity RNA genome is >9000 nucleotides (nt) long and contains strong RNA secondary structures [38]. Thus far, only ASOs targeting the 350-nt region at the 5’-terminus of HCV RNA, which contains the internal ribosome entry site (IRES), are efficient inhibitors of viral replication [15,39–46]. Hence, HCV RNA represents an important but difficult target for ASOs, and it may enable the determination of the positive and negative impacts of ASO modifications.

Here, we demonstrate that the incorporation of 8-oxo-dG residues destabilizes not only ASO:DNA but also ASO:RNA duplexes. These modifications also slow down the formation of all-DNA ASO:RNA duplexes, but they have little effect on the formation of duplexes between an LNA/DNA gapmer ASO and its target RNA. Furthermore, the presence of 8-oxo-dG residues does not have a negative impact on the efficiency of RNase H-mediated cleavage of the RNA target. Simultaneously, this modification alters the specificity of RNase H cleavage and increases the stability of ASOs in human serum. RNA interference (RNAi)-guided target site selection was used to identify novel sites in the coding region of the HCV genome that could be efficiently targeted with small interfering RNAs (siRNAs) and ASOs. The incorporation of 8-oxo-dG residues into the DNA region of an LNA/DNA gapmer oligonucleotide that targets such sites led to the development of ASOs that exhibit this novel mechanism of antisense action.

## Materials and Methods

### Oligonucleotides and modified oligonucleotides

siRNAs targeting the coding region of the HCV genome were designed using an algorithm that was developed in-house and were obtained from *Sigma-Aldrich* (USA). Validated controls, including non-targeting siRNAs (AM4611 or AM4635) and a combination of siRNAs against firefly luciferase (Luc) [47] (AM4629), were obtained from *Life Technologies* (USA). If not stated otherwise, the non-modified all-DNA oligonucleotides (hereafter designated as “D”), modified all-DNA oligonucleotides (designated as “DM”), LNA/DNA gapmers (designated as “LD”) and LNA/DNA gapmers with modified nucleobases (designated as “LDM”) were synthesized by *Exiqon A/S* (Denmark). All the obtained oligonucleotides were dissolved in sterile water, aliquoted and stored at -85°C.

The batches of modified oligonucleotides obtained from different commercial providers were of variable quality. Accordingly, they failed to produce consistent and reproducible results in biological assays. For these reasons, multi-step quality control and purification protocols were developed. The quality of each oligonucleotide batch was independently verified in-house by RP-HPLC analysis using an HPLC LC20-A Prominence system (*Shimadzu*, Japan) equipped with an SPD-M20A absorbance detector. A Phenomenex Luna 5 μm C18 100A column (250x4.6 mm, SecurityGuard C18 4x3 mm precolumn) was temperature-regulated at 45°C and operated at a flow rate of 1 ml/min. The oligonucleotides were eluted using a methanol gradient in 0.02 M ammonium phosphate buffer, pH 7.0 [48]. For chromatogram analysis, Shimadzu LCsolution software was used. For preparative purification, inorganic phosphate was removed from the RP-HPLC fractions by the IE-HPLC method using LiClO_4_ as the eluent. The fractions were precipitated and washed with acetone. Depending on the downstream application, the oligonucleotide preparations were re-precipitated using ethanol/ammonium acetate (pH 7.0) or ethanol/sodium acetate. The oligonucleotides used in the subsequent analyses contained only trace quantities of impurities.

Mass spectrometric analysis of oligonucleotides was performed as described previously [48] using a Bruker Daltonics Autoflex instrument. Briefly, the matrix solution consisted of 7.8 mg/ml 2,4,6-trihydroxyacetophenone and 12 mM diammonium citrate in acetonitrile/water (1:1). An aliquot of the concentrated chromatographic fraction (or original preparation of oligonucleotide) was mixed with the same volume of matrix solution. One microliter of the resultant mixture was deposited on the target and dried in the air. The samples were analyzed in negative ion mode with the linear configuration.

### Melting curve analysis using Förster Resonance Energy Transfer (FRET)

Melting curve data were obtained using FRET as described in detail by You and coworkers [49]. Briefly, DNA or RNA oligonucleotides labeled with Cy3 or TYE563 at the 3’-end (hereafter designated as “target”) were purchased from *DNA Technology* (Denmark) or *Sigma-Aldrich* (USA). Complementary oligonucleotides (hereafter designated as “probes”) were labeled in-house with FITC at the 5’-end or purchased with a 5’ FAM label from *Exiqon A/S* (Denmark). Hybridization was quantified by FRET between the FITC and Cy3 labels or the FAM and TYE563 labels (detected as a decrease in FITC or FAM fluorescence) when they were brought in close proximity due to the formation of a duplex between the probe and its target. The target oligonucleotides were used at concentrations of 25, 50, 100, 200 and 400 nM, and the probe was always used at a concentration of 50 nM. The probe-target interactions were measured in a 384-well optical plate in a volume of 20 μl in buffer containing 150 mM NaCl and 50 mM Tris-HCl (pH 8.0) using an ABI7900HT Real-Time PCR instrument (*Life Technologies*, USA). The temperature was rapidly increased to 95°C, and the complexes were allowed to melt for 10 min. Then, the temperature was decreased to 5°C over 45 min, stabilized for 5 min and increased slowly (over 45 min) to 95°C. The Tm values were calculated from the obtained melting curves.

### Preparation of ASO:RNA duplexes

An ssRNA with the sequence 5’-GGC UUU ACC GGC GAU UUC GAC UCA GUG AUC GAC UGC A-3’ (*Exiqon A/S*, Denmark) was labeled at the 5´ terminus using γ-^33^P ATP (*Perkin Elmer*, USA) and phage T4 polynucleotide kinase (*Thermo Scientific*, USA) according to the manufacturers’ protocols, purified, and dissolved in buffer containing 10 mM HEPES, pH 7.2, and 20 mM KCl. The labeled RNA was mixed with partially complementary D4676, DM4676, LD4676, LDM4676 or MixLD4676 oligonucleotides (Table 1). The obtained mixtures were heated to 95°C and allowed to slowly cool to 35°C. The appropriate volume of 5x non-denaturing loading buffer containing 50% glycerol, 0.1% bromophenol blue and 0.1% xylene cyanol was added to the samples, after which ASO:RNA duplexes were purified and quantified as described previously [50].

**Table 1:** Sequences of DNA and LNA/DNA gapmer oligonucleotides.

Compound	Sequence (5’->3’) and modifications
D4676	NH2-ATC ACT GAG TCG AAA TCG CCG
D4676inv	NH2-GCC GCT AAA GCT GAG TCA CTA
LD4676	NH2-+A+T+C +A+CT GAG TCG AAA T+C+G +C+C+G
LD4676inv	NH2-+G+C+C +G+CT AAA GCT GAG T+C+A +C+T+A
DM4676	NH2-ATC ACT YAY TCY AAA TCG CCG
LDM3570	NH2-+A+T+G +A+TA GAX AGT XXA A+C+A +C+A+C
LDM3570inv	NH2-+C+A+C +A+CA AXX TGA XAG AT+A +G+T+A
LDM4676	NH2-+A+T+C +A+CT YAY TCY AAA T+C+G +C+C+G
LDM4676inv	NH2-+G+C+C +G+CT AAA YCT YAY T+C+A +C+T+A
MixLD4676	NH2-+AT+C AC+T GAG +TCG +AAA +TC+G C+C+G

NH2 = 5’ amino modifier C6. This group was present only in the oligonucleotides used for melting curve determination and for the analysis of delivery into cells. + = prefix for LNA; X = 5-OH-dC; Y = 8-oxo-dG.

To analyze the spontaneous formation of duplexes between target ssRNA and ASOs, 5 fmol of ^33^P-labeled ssRNA was mixed with 50 fmol of D4676, DM4676, LD4676 or LDM4676 (Table 1) in buffer containing 10 mM HEPES, pH 7.2, and 20 mM KCl [50]. Samples were collected immediately (0 time point) or after incubation for 0.5, 1, 2, 4 or 8 h at 37°C. The appropriate volume of 5x non-denaturing loading buffer was added, and the samples were analyzed by PAGE in 15% native gels. The gels were dried, exposed to a storage phosphor screen and visualized using a Typhoon Trio scanner (*GE Healthcare*, UK).

### *In vitro* RNase H assay

Target RNA (designated as FR3131), consisting of 269 nt from the 5’ end of the HCV genome and the region spanning positions 3081 to 5943, was synthesized *in vitro* using an mMESSAGE mMACHINE T7 Transcription Kit (*Life Technologies*, USA) according to the manufacturer’s instructions. RNase H assays were performed as described by Kurreck and co-authors [19]. Briefly, the reaction mixture contained 1x RNase H buffer, 0.5 U of bacterial RNase H (*Thermo Scientific*, USA), 5 pmol of ASO and 500 ng of FR3131 RNA. The reaction mixture was incubated at 37°C for 0, 1, 5, 10, 20 or 60 min. At each time point, a 10 μl aliquot was collected. The reaction was stopped by adding 10 μl of 2x stop buffer (50 mM EDTA and 1% SDS) and subsequent heating to 95°C for 2 min. The reaction products were separated on a 0.8% TAE agarose gel and detected with ethidium bromide staining.

The kinetics of ASO:RNA duplex cleavage were analyzed using pre-formed ASO:RNA duplexes. Briefly, 1 fmol of the labeled duplexes was mixed with 0.05 U of RNase H in 1x RNase H buffer, and the reaction was performed at 37°C. Aliquots were collected immediately after mixing the substrate and enzyme (0 time point) or after incubation for 10 s or 0.5, 1, 5, 10 or 20 min. The reactions were stopped by adding an equal volume of 2x stop buffer. The samples were then heated to 95°C for 2 min, cooled rapidly on ice and separated by PAGE in native 15% gels. The gels were dried, exposed to a storage phosphor screen and visualized using a Typhoon Trio scanner (*GE Healthcare*, UK). Quantitative analyses were performed using ImageQuant TL Software (*GE Healthcare*, UK).

### Analysis of the stability of ASOs in human serum

To analyze the stability of D4676, DM4676, LD4676, and LDM4676 (Table 1) in human serum, these compounds were labeled with ^33^P as described above for RNA oligonucleotides. Five fmol of each ^33^P-labeled ASO was incubated in human serum (Human Serum, Off the clot, Type AB; *PAA*, Germany) at 37°C. Aliquots were collected immediately after preparation of the mixtures (0 time point) or after incubation for 0.25, 0.5, 1, 2, 4 or 6 h. Next, 2x stop solution was added to each aliquot. The full-length oligonucleotides and their degradation products were separated and analyzed as described above. The average half-lives of each type of ASO were obtained from three independent experiments by fitting the obtained values to an exponential decay function.

### HCV replicon cells

**Fig 2:**
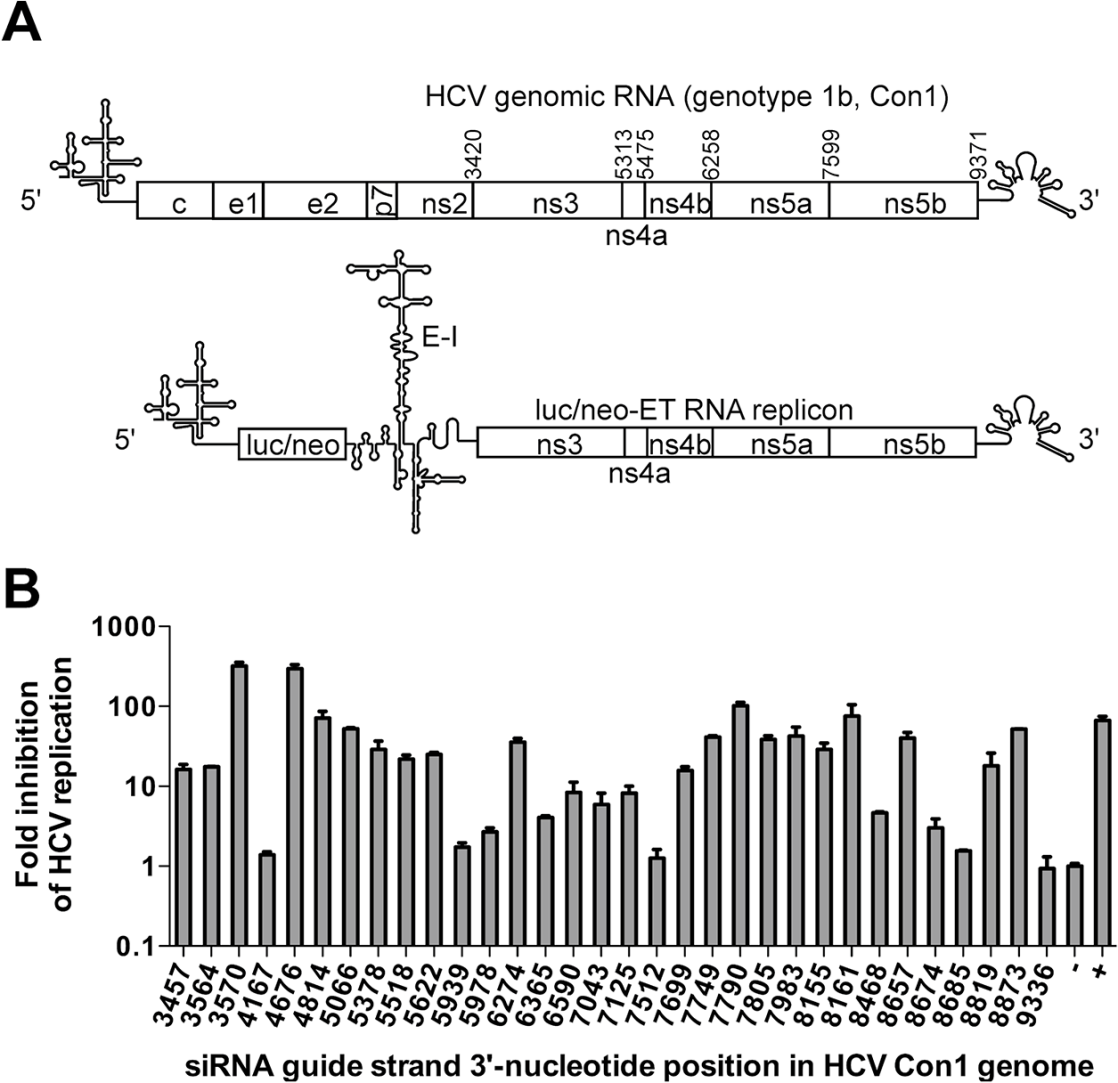
RNAi-guided oligonucleotide target-site selection in the coding region of HCV RNA. **(A)** Schematic of the HCV genome and the luc/neo-ET (I_389_/NS3-3’/LucUbiNeo-ET) replicon. The numbers above the HCV genomic RNA indicate the positions of the start codons for the non-structural proteins NS3-NS5B. Luc/neo, firefly luciferase/neomycin phosphotransferase cassette; E-I, encephalomyocarditis virus IRES element. **(B)** Inhibitory effects of thirty-two different siRNAs targeting the NS3-NS5B region of the luc/neo-ET replicon. The siRNAs (Table 3, guide strands are indicated on the *x*-axis; “+”, combination of control siRNAs targeting the Luc reporter gene) were transfected into Huh-luc/neo-ET cells at a concentration of 100 nM. At 48 h p.t., the total protein content and Luc activities in cell lysates were determined. The Luc activities were first normalized to total protein content; next, the obtained values were normalized to the value obtained for control cells transfected with non-targeting negative control siRNA (designated as “-”), which was set to 1. The *y*-axis indicates the fold inhibition of HCV replication achieved using the corresponding siRNAs (note the logarithmic scale). The error bars represent the standard deviation of three independent experiments.

Huh-luc/neo-ET cells, which harbor the I389/NS3-3’/LucUbiNeo-ET replicon of HCV genotype 1b (Con1 isolate) (Fig 2A), and replicon-free Huh7-cure cells were obtained from *ReBlikon GmbH* (Germany). The cells were maintained in Dulbecco’s modified Eagle’s medium supplemented with penicillin, streptomycin, 0.5 mg/ml G418, 10% fetal calf serum and 2 mM L-glutamine (*GE Healthcare*, UK).

A variant replicon that encodes for NS3 with Thr54Ala mutation, was constructed using site-directed mutagenesis and designated I389/NS3-3’/LucUbiNeo-ET-T54A. The corresponding cell line, designated Huh-luc/neo-ET-3570mut, was obtained by electroporation [51] of Huh7-cure cells with the corresponding *in vitro-*transcribed RNAs and selection of antibiotic-resistant colonies in the presence of 0.5 mg/ml G418 (*Invivogen*, USA). The preservation of the introduced mutation was verified as follows. Total RNA was extracted from Huh-luc/neo-ET-3570mut cell line using an RNeasy Mini Kit (Qiagen). Reverse transcription was carried out using a First-Strand cDNA Synthesis kit (Thermo Scientific). HCV-specific cDNA fragment containing the mutation site was PCR-amplified using of primers flanking the mutated region. Obtained PCR products were purified and sequenced using Sanger sequencing.

### Transfection of cells with siRNAs or ASOs

For RNAi-guided screening, Huh-luc/neo-ET cells were reverse-transfected with siRNAs (100 nM each) using Lipofectamine RNAiMAX reagent (*Life Technologies*, USA). Luc activity was measured 48 h post-transfection (p.t.) using reagents and protocols from *Promega* (USA). The total protein content in the cell lysates was measured by Bradford micro-assay (*Bio-Rad*, USA).

Lipofectamine 2000, Lipofectamine RNAiMAX, Lipofectamine LTX (*Life Technologies*, USA), DOTAP, FuGENE HD (*Roche*, Switzerland) and TurboFect (*Thermo Scientific*, USA) reagents were used to optimize the transfection of Huh-luc/neo-ET cells. Various amounts of these reagents and forward- or reverse-transfection protocols were used to deliver ASOs (Table 1) conjugated to Alexa Fluor 568 into the cells. The transfection efficiencies were analyzed using an LSRII flow cytometer (*BD Biosciences*, USA). Cytotoxic effects (or lack thereof) were observed using a Nikon Eclipse confocal microscope (*Nikon*, Japan).

### Quantitation of the inhibitory effects of ASOs

Cells were collected and lysed at selected time points. The total protein content in the lysate and Luc activity were measured. For the normalization of HCV replication, which is proportional to Luc activity, the following calculations were performed. First, to enable the comparison of the average Luc activity per living cell, the total protein content of the cells was used to normalize the Luc activity as previously reported [51]. Thus, the HCV replication signal was expressed as relative light units per microgram of protein (RLU/μg protein). Second, the obtained normalized Luc values were divided by those obtained for the negative controls: “-” siRNA- or mock-transfected cells. The averages and standard deviations of seven independent experiments were obtained. Subsequently, the dimensionless average values were fitted with a four-parameter dose-response equation (variable slope model) using Prism 5 (*GraphPad Software*, *Inc*., San Diego, CA, USA) to estimate the effective concentration 50 (EC_50_) values.

## Results

### Thermal stability of the all-DNA ASO:RNA duplex is reduced upon incorporation of 8-oxo-dG residues

8-oxo-dG residues have been reported to destabilize ASO:DNA duplexes [27,29,30]. However, ASOs are generally used to target RNA rather than DNA molecules. To analyze the effects of 8-oxo-dG residues on the binding of ASOs to DNA and RNA molecules, a set of all-DNA oligonucleotides was prepared in which none, one, or two of the centrally located dG residues were substituted with 8-oxo-dG residues. For comparison, a set of ASOs containing 5-OH-dC residues was prepared because of the similarity to 8-oxo-dG; the 5-OH-dC minor tautomeric form (Fig 1A) was predicted to have abnormally strong bonding to dG residues [33]. Nevertheless, the introduction of 5-OH-dC residues simultaneously into ASO and target DNA also results in decreased ASO:DNA duplex stability [28]. All the oligonucleotides were 21 nt long and had identical sequences (Table 2). Duplex formation between these oligonucleotides and their targets was monitored by FRET. In this setup, any difference in the Tm of the formed duplexes is attributable to the presence of the 8-oxo-dG or 5-OH-dC modifications.

**Table 2:** Sequences of oligonucleotides used to determine the melting temperatures by FRET.

Compound	Sequence (5’->3’) and modifications
Target RNA-Cy3	GAU UCU GAU GAC UCA UUU CUU-Cy3
Target DNA-Cy3	GAT TCT GAT GAC TCA TTT CTT-Cy3
FITC-C	FITC-AAG AAA TGA GTC ATC AGA ATC
FITC-X	FITC-AAG AAA TGA GTX ATC AGA ATC
FITC-XX	FITC-AAG AAA TGA GTX ATX AGA ATC
FITC-Y	FITC-AAG AAA TYA GTC ATC AGA ATC
FITC-YY	FITC-AAG AAA TYA YTC ATC AGA ATC
Target RNA-TYE563	CGG CGA UUU CGA CUC AGU GAU-TYE563
Target DNA-TYE563	CGG CGA TTT CGA CTC AGT GAT-TYE563
FAM-D4676	FAM-ATC ACT GAG TCG AAA TCG CCG
FAM-LD4676	FAM-+A+T+C +A+CT GAG TCG AAA T+C+G +C+C+G
FAM-LDM4676	FAM-+A+T+C +A+CT YAY TCY AAA T+C+G +C+C+G

NH2 = 5’ amino modifier C6; + = prefix for LNA; X = 5-OH-dC; Y = 8-oxo-dG.

When the 8-oxo-dG modification (FITC-Y) was introduced, the Tm values of both the ASO:DNA and ASO:RNA duplexes were reduced by ~1.6–1.8°C compared to those of the duplexes formed by control oligonucleotides. Increasing the number of 8-oxo-dG modifications (FITC-YY) resulted in further reduction of the Tm by ~1.6°C for ASO:DNA and by ~2.5°C for ASO:RNA duplexes. Thus, destabilization resulting from the insertion of 8-oxo-dG modifications occurs not only for ASO:DNA duplexes (Fig 1B) but also for ASO:RNA duplexes (Fig 1C). In contrast, duplexes formed by oligonucleotides with one or two 5-OH-dC residues had nearly the same Tm as duplexes formed by unmodified control oligonucleotides (Fig 1B and 1C). Thus, the introduction of the 5-OH-dC modification into an ASO alone had virtually no effect on the stability of duplexes formed with DNA or RNA.

### RNAi-guided selection reveals potential ASO target sites in the coding region of HCV RNA

Known targets for ASOs are located in a 350-nt region at the 5’-terminus of HCV RNA. Clearly, targeting a region that comprises less than four percent of the virus genome is a bottleneck that hinders the development of the most efficient ASOs. RNAi technology was used to search for highly accessible target sites in the HCV coding region. Twenty-eight different siRNAs targeting the HCV genome were designed (Table 3). Each siRNA had a 19-nt duplex region with 2-nt 3’-overhangs [52]. In addition, four siRNAs (3564, 7749, 7805, and 7983) that were previously reported to efficiently inhibit HCV replication [53,54] were used for comparison. Non-targeting siRNAs (AM4611 or AM4635) and a combination of siRNAs against a sequence encoding Luc marker (AM4629) [47] were used as negative and positive controls, respectively.

**Table 3:** Sequences of oligonucleotides in siRNA duplexes.

Position^a^	Antisense strand (5'->3')	Sense siRNA strand (5'->3')
3457	UAG UGA UGA UGC AGC CAA GUA	C UUG GCU GCA UCA UCA CUA GC
3564	GAC AGU CCA ACA CAC GCC AUU	U GGC GUG UGU UGG ACU GUC UA
3570	AUG AUA GAC AGU CCA ACA CAC	G UGU UGG ACU GUC UAU CAU GG
4167	UAC CCC GGU UCU GAU GUU AGG	U AAC AUC AGA ACC GGG GUA AG
4676	AUC ACU GAG UCG AAA UCG CCG	G CGA UUU CGA CUC AGU GAU CG
4814	AUG CCC AUC CUG CCC CUA CCA	G UAG GGG CAG GAU GGG CAU UU
5066	UUG UCU CCU GCC UGC UUA GUC	C UAA GCA GGC AGG AGA CAA CU
5378	AUC CUG CCC ACA AUG ACC ACG	U GGU CAU UGU GGG CAG GAU CA
5518	UUG CCU UCU GUU UGA AUU GUU	C AAU UCA AAC AGA AGG CAA UC
5622	AUU CCA CAU AUG CUU CGC CCA	G GCG AAG CAU AUG UGG AAU UU
5939	AUC UCG CCG CUC AUG ACC UUA	A GGU CAU GAG CGG CGA GAU GC
5978	AUA GCA GGG AGU AGG UUA ACC	U UAA CCU ACU CCC UGC UAU CC
6274	AUA UCC AAU CCC AAA CAU CUC	G AUG UUU GGG AUU GGA UAU GC
6365	UAC CCA CGU UGA CAU GAG AAG	U CUC AUG UCA ACG UGG GUA CA
6590	UAC UCC UCA GCA GCC ACC CGC	G GGU GGC UGC UGA GGA GUA CG
7043	AUG UUC CCG CCC AUC UCC UGC	A GGA GAU GGG CGG GAA CAU CA
7125	UAC UUC CCU CUC AUC CUC CUC	G GAG GAU GAG AGG GAA GUA UC
7512	AUC CCC CGG CUC CCC CUC AAG	U GAG GGG GAG CCG GGG GAU CC
7699	UUG UAG CAU AGA CCA AGU UGU	A ACU UGG UCU AUG CUA CAA CA
7749	CAG UCU GUC AAA GGU GAC CUU	G GUC ACC UUU GAC AGA CUG CA
7790	AUC UCC UUG AGC ACG UCC CGG	G GGA CGU GCU CAA GGA GAU GA
7805	GAC GCC UUC GCC UUC AUC UCC	A GAU GAA GGC GAA GGC GUC CA
7983	GUC AAU UGG UGU CUC AGU GUC	C ACU GAG ACA CCA AUU GAC AC
8155	AUC CGU AUG AAG AGC CCA UCA	A UGG GCU CUU CAU ACG GAU UC
8161	AUU GGA AUC CGU AUG AAG AGC	U CUU CAU ACG GAU UCC AAU AC
8468	AAG UAA CAU GUG AGG GUA UUA	A UAC CCU CAC AUG UUA CUU GA
8657	AAG UCG UAU UCU GGU UUG GGC	C CAA ACC AGA AUA CGA CUU GG
8674	AUG AUG UUA UCA ACU CCA AGU	U UGG AGU UGA UAA CAU CAU GC
8685	AUU GGA GGA GCA UGA UGU UAU	A ACA UCA UGC UCC UCC AAU GU
8819	AUG AUG AUG UUG CCU AGC CAG	G GCU AGG CAA CAU CAU CAU GU
8873	AUG GAG AAG AAA UGA GUC AUC	U GAC UCA UUU CUU CUC CAU CC
9336	AUA GAU GCC UAC CCC UAC AGA	U GUA GGG GUA GGC AUC UAU CU

^a^ Position refers to the terminal 3’-end nucleotide position of the siRNA antisense strand in the HCV Con1 genome (GenBank accession number: AJ238799).

The level of Luc activity in Huh-luc/neo-ET cells is directly proportional to the copy number and replication efficiency of the HCV subgenomic replicon, making it an efficient tool for analyzing the anti-HCV efficiencies of the obtained siRNAs [55,56]. At a concentration of 100 nM, the majority of the designed HCV-specific siRNAs induced less of an effect that the positive controls (Fig 2B). Moreover, similarly to the negative control siRNA, several siRNAs (4167, 7512, 8685, and 9336) did not have any effect on HCV replication. The effects of three siRNAs (4814, 7790 and 8161) were comparable to those of the positive controls (70- to 100-fold inhibition), and two siRNAs (3570 and 4676) were more potent (approximately 300-fold inhibition).

As the high inhibitory potential of an siRNA indicates the accessibility of the corresponding target sites, it was concluded that RNAi-guided screening enabled the selection of several potential ASO target sites in the HCV coding region. However, an all-DNA ASO based on the sequence of the guide strand of siRNA 4676 was essentially unable to suppress HCV replication. Therefore, HCV-specific 21-mer LNA/DNA gapmer oligonucleotides that contained five LNA monomers at each end and three modified residues in the DNA region were designed (Tables 1 and 2). As the target site of siRNA 4676 contained three C-residues in its central region, it was targeted by ASOs containing three 8-oxo-dG nucleotides. In contrast, the target site for siRNA 3570 contained three G-residues, and hence, a control ASO was generated that contained three 5-OH-dC residues (Table 1).

### Inhibitory efficiency of modified ASOs is reduced by point mutation in the target site

**Fig 3:**
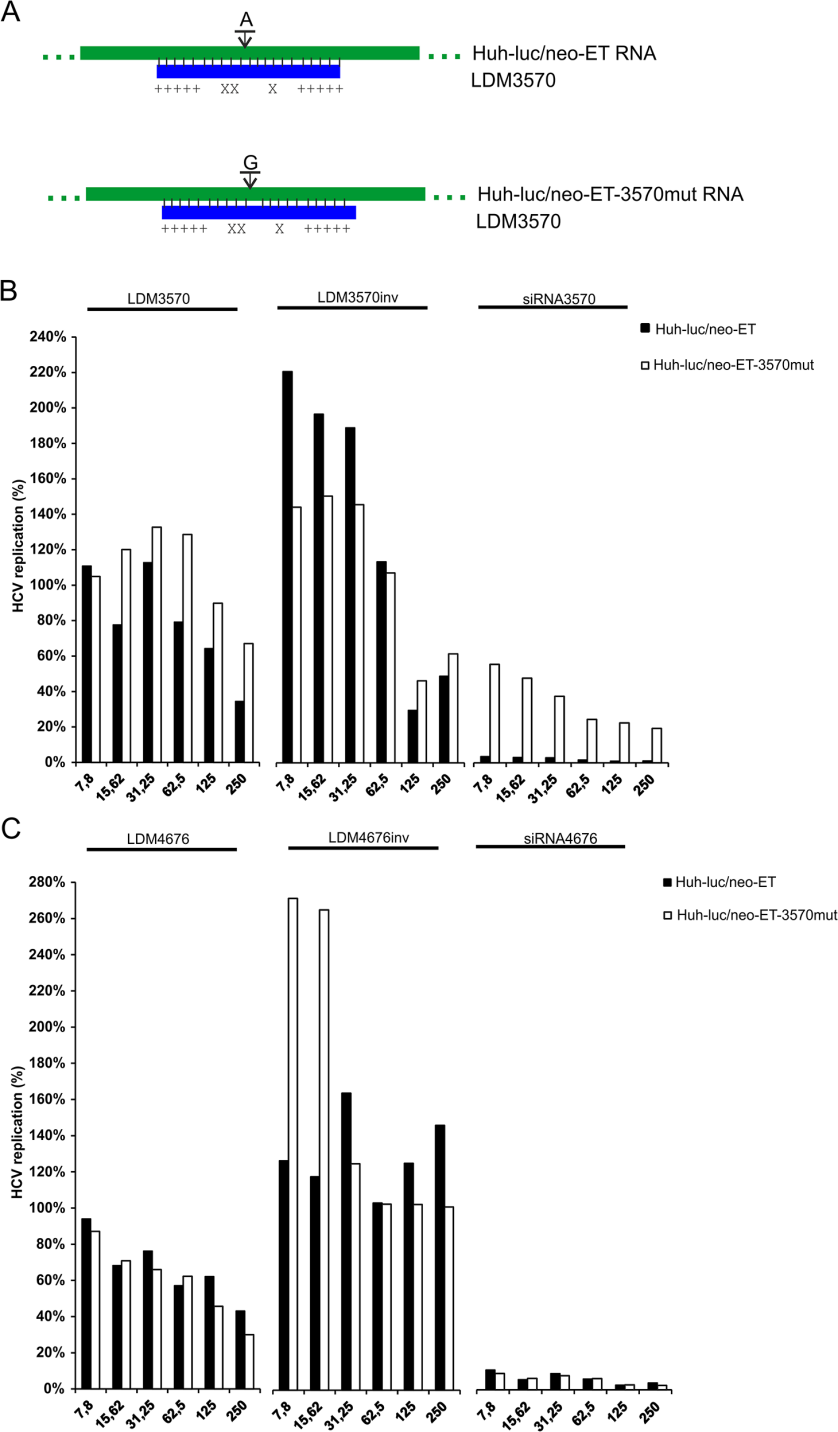
Modified LNA/DNA gapmer oligonucleotide potency is reduced by point mutation in its target site. **(A)** Schematic of the native (above) and mutant (below) siRNA 3570 target sites in the HCV replicon bound to LDM3570 (X, 5-OH-dC residue; +, LNA sugar base). **(B, C)** Huh-luc/neo-ET (black bars) and Huh-luc/neo-ET-3570mut (white bars) cells were transfected with increasing concentrations (*x*-axis) of LDM3570, LDM3570inv and siRNA 3570 **(B)** or LDM4676, LDM4676inv and siRNA 4676 **(C)**. The HCV replication values (*y*-axis) were calculated as described for Fig 2B. The obtained values were subsequently normalized to those from mock-transfected control cells, which was set to 100%. Each panel represents data from one of two reproducible independent experiments.

The inhibitory effects of siRNAs and, to lesser extent, ASOs are reduced by point mutations in their target sites. Mutations at distant sites (unless they modify the higher-order structure of the target region) have no such effect. The only mutation in the selected target sites, for which the viability of the mutant replicon has been previously demonstrated, is located in the target site of siRNA 3570 and results in a Thr54→Ala change in NS3 [57]. Therefore, this mutation was introduced into the HCV replicon that was used to generate a Huh-luc/neo-ET-3570mut cell line (Fig 3A). As the central region of the target site of siRNA 3570 contains only two C-residues, an ASO similar to LDM4676 (which contains three 8-oxo-dG residues; Table 1) could not be designed against this site. Therefore, a control LNA/DNA gapmer containing three 5-OH-dC residues (LDM3570; Fig 3A) was used instead. Oligonucleotides with inverted sequences (LDM4676inv and LDM3570inv; Table 1) were used as controls.

Huh-luc/neo-ET and Huh-luc/neo-ET-3570mut cells were transfected with different concentrations of siRNA 3570, siRNA 4676, LDM4676, LDM4676inv, LDM3570 and LDM3570inv. A point mutation in HCV RNA that changes the classical A:U base pair in the siRNA guide-strand:target-RNA duplex to the G:U wobble base pair resulted in a marked (10-fold) decrease in the inhibitory efficiency of siRNA 3570 (Fig 3B). As expected, this mutation did not alter the inhibitory efficiency of siRNA 4676 or LDM4676. Transfecting cells with different amounts of LDM4676inv resulted in increased HCV replication regardless of the presence or absence of the mutation in the siRNA 3570 target site (Fig 3C). In contrast to LDM4676, the inhibitory activity of LDM3570 was clearly reduced by the mutation (Fig 3B). At concentrations up to 62.5 nM, the control oligonucleotide LDM3570inv was unable to suppress HCV replication, and its effects (if any) were not diminished by the mutation in the siRNA 3570 target site (Fig 3B). Therefore it can be concluded that at these concentrations the potency of LDM3570 was indeed specifically reduced by mutation of its target site. These findings are consistent with the antisense mechanism of action of modified oligonucleotides; furthermore, the data indicate that the observed suppression of HCV replication was not caused by side effects of the ASOs. However, at concentrations 125 or 250 nM, LDM3570inv exhibited toxicity and inhibited HCV replication (Fig 3B).

### Incorporation of 8-oxo-dG residues into LNA/DNA gapmer oligonucleotides has no negative impact on their antisense potency

**Fig 4:**
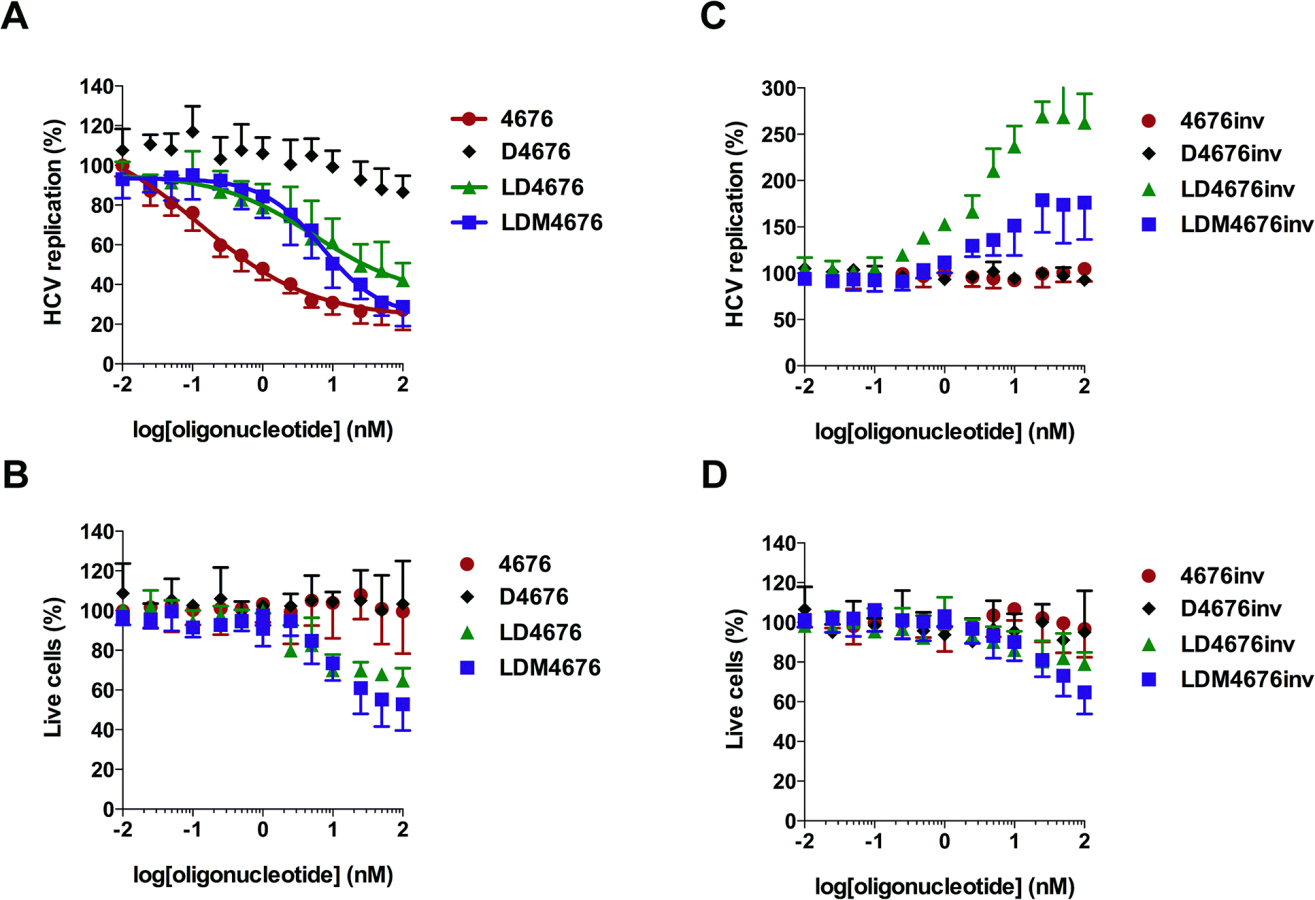
Effects of 8-oxo-dG residues on antisense potency and off-target effects of LNA/DNA gapmer oligonucleotides. Huh-luc/neo-ET cells were transfected with increasing concentrations (*x*-axis) of various oligonucleotides (Table 1) targeting the 4676 site **(A, B)** or with inverted non-targeting control oligonucleotides **(C, D)**. The error bars represent the standard deviation of seven independent experiments. **(A, C)** The effects of the oligonucleotides on HCV replication are shown on the *y*-axis. Transfection and normalization of Luc activity were performed as described for Fig 2B. The obtained values were subsequently normalized to those from mock-transfected control cells, which were set to 100%. The values for siRNA 4676, LD4676, and LDM4676 were fitted with a four-parameter dose-response equation (variable slope model); estimated EC_50_ values are shown in Table 4. **(B, D)** Percentage of living cells in transfected cell cultures. The total protein content in the lysates of transfected Huh-luc/neo-ET cells was normalized to that of mock-transfected cells (set to 100%) to obtain the percentage of living cells (*y*-axis).

At the highest concentrations, LDM4676 and LDM3570 visibly damaged the transfected cells. Although these ASOs lack the nucleotide sequence descriptors (TCC and TGC motifs) characteristic of hepatotoxic LNA/DNA gapmer oligonucleotides [58], cytotoxicity may result from additional factors and their combinations. These factors include the transfection protocol, the presence of modified nucleobases and the cytotoxicity of the transfection reagent. As cytotoxicity represents an obstacle for more detailed studies, more suitable transfection methods were sought. Huh7 cells and their derivatives were difficult to transfect with DNA or LNA/DNA gapmer oligonucleotides without causing cell damage. Therefore, the results obtained using different amounts of six transfection reagents (see Materials and Methods) and reverse- or direct-transfection protocols were compared. Based on the results of this comparison, an optimized Lipofectamine 2000-mediated reverse-transfection protocol was selected that enabled the delivery of siRNA and ASOs into 70–75% of the cells. Further increases in transfection efficiency required higher amounts of transfection reagent, which resulted in decreased viability of transfected cells. Thus, in subsequent experiments, the reduction of HCV replication to 25–30% of its original level roughly corresponded to complete suppression of HCV replication in every siRNA- or ASO-transfected cell. Even using the selected protocol, higher concentrations of LD4676, LDM4676 and LDM4676inv exhibited some cytotoxicity, as determined by measuring the total protein content in the lysates of transfected cells (Fig 4B–4D). Cell viability measurements using the xCELLigence system (*ACEA Biosciences*, USA) revealed the same trend. Therefore, as in all previous experiments (Figs 2B, 3B and 3C), the replication signal (Luc activity) was normalized to the total protein content of the cell lysate (essentially, to the number of living cells). The quantitative evaluation by four-parameter dose-response curve fitting (variable slope model) of such values obtained in seven independent experiments is shown in Fig 4A, and the obtained EC_50_ values for different ASOs are summarized in Table 4.

**Table 4:** EC_50_, CI and R^2^ values for LD4676, LDM4676, and siRNA 4676 estimated from seven independent experiments.

ON/siRNA	EC_50_ (nM)	CI (nM)	R^2^
D4676	ND	ND	ND
LD4676	5.5	0.9–32.9	0.77
LDM4676	7.1	4.0–12.5	0.87
siRNA 4676	0.13	0.03–0.5	0.95

CI = 95% confidence interval; R^2^ = goodness of a four-parametric nonlinear regression curve fit; ND = not determined.

The obtained data revealed that D4676 did not considerably reduce HCV RNA replication, confirming a previous observation that the all-DNA ASOs are not efficient inhibitors. siRNA 4676 had an EC_50_ of 0.13 nM, whereas the EC_50_ values for LD4676 and LDM4676 were approximately 50-fold higher (Table 4). Interestingly, despite the roughly equal mean EC_50_ values of LD4676 and LDM4676, the shape of the LD4676 inhibitory curve at higher concentrations was linear rather than non-linear, which was also evident by the goodness of fit with the variable slope non-linear regression curve (Table 4). Consequently, at the highest concentrations, LDM4676 inhibited the HCV replication signal to a approximately 27% residual level. As approximately 25% inhibition was achieved with siRNA 4676 (Fig 4A), which is capable of nearly completely inhibiting HCV replication in positively transfected cells (Fig 2B), it was calculated that 100 nM LDM4676 suppressed HCV replication by at least 95% in ASO-transfected cells. In contrast, the residual HCV replication level in the presence of the highest concentration of LD4676 was 42% (Fig 4A), indicating that HCV replication in ASO-transfected cells was inhibited by no more than 80%. To confirm that the observed inhibitory effects (Fig 4A) were sequence-specific, control oligonucleotides with inverted sequences (Table 1) were transfected into Huh-luc/neo-ET cells. Importantly, despite an observation of mild cytotoxicity at the highest concentrations, none of the control oligonucleotides inhibited HCV replication (Fig 4C and 4D); these data indicated that the observed effects of the ASOs targeting the siRNA 4676 site were sequence-specific. These results also demonstrated that under certain conditions, LDM4676 might be a more efficient inhibitor of HCV RNA replication than LD4676. However, in general, the incorporation of 8-oxo-dG residues did not result in significant gains in antisense potency in a cell-based HCV replication assay.

### 8-oxo-dG residues reduce the Tm of the LNA/DNA gapmer ASO:RNA duplex but have little effect on duplex formation

The Tm values of the LDM4676:DNA and LDM4676:RNA duplexes were determined and compared to the Tm values of duplexes composed of non-modified all-DNA ASO (D4676) or LNA/DNA gapmer ASO (LD4676) (Table 1).

**Fig 5:**
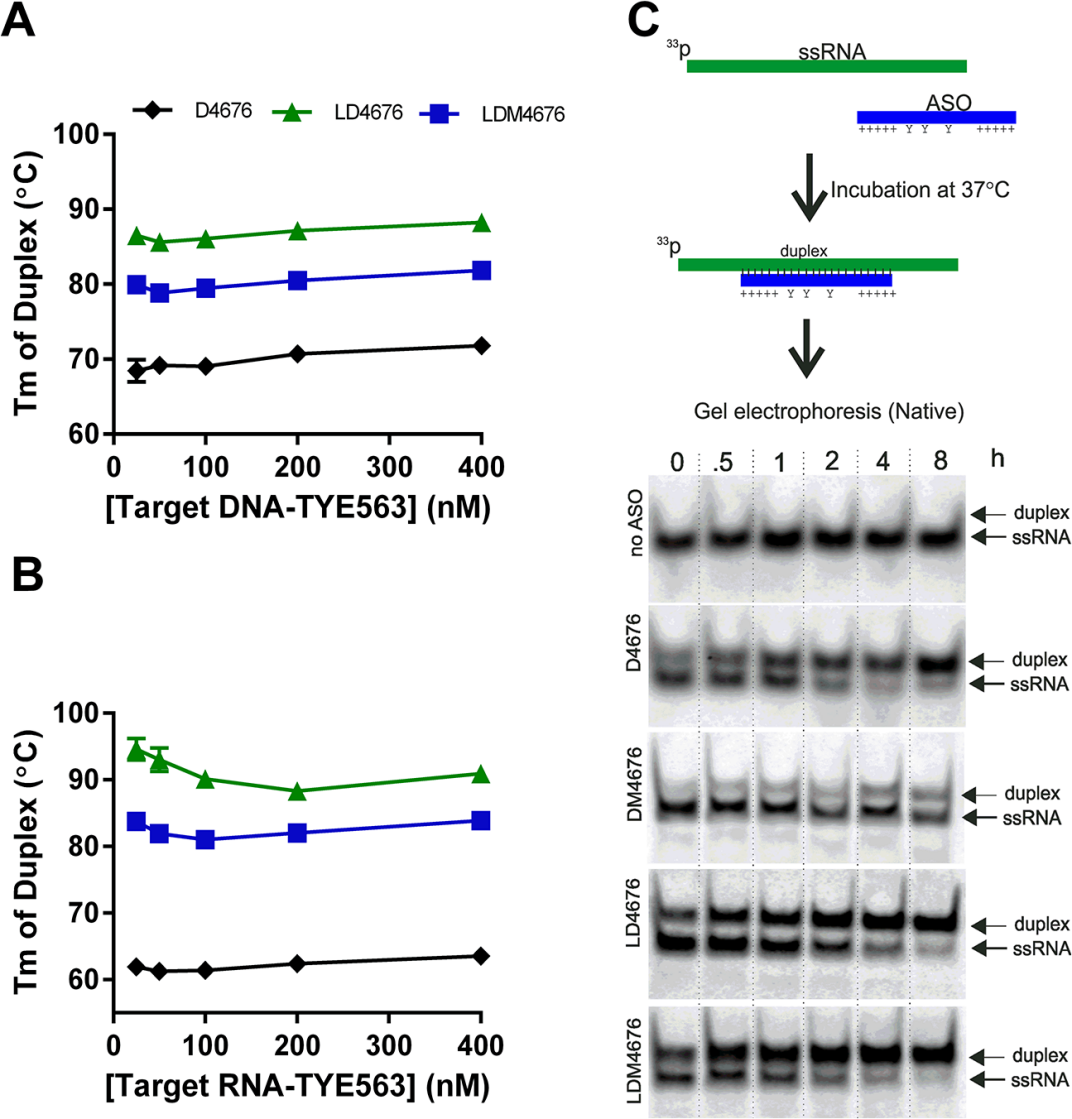
8-oxo-dG residues reduce the Tm of duplexes between LNA/DNA gapmers and their targets. The effects of 8-oxo-dG residues on the Tm of LNA/DNA gapmer ASO:DNA **(A)** and LNA/DNA gapmer ASO:RNA (**B**) duplexes were measured by FRET. Target DNA or RNA oligonucleotides (Table 2) were labeled with TYE563 at the 3’-end; the D4676, LD4676 and LDM4676 probes had FAM at the 5’-end. The measurements were performed, and the data are presented as described for Fig 1 **(C)**. The effect of 8-oxo-dG residues on ASO:RNA duplex formation. Upper: schematic of the experimental setup. Applicable for some ASOs: Y, 8-oxo-dG residue; +, LNA sugar base. Lower: the ^33^P-labeled 37-nt ssRNA target was mixed with the indicated ASOs. The samples were collected immediately (“0”) or after incubation at 37°C for the indicated times. The obtained probes were resolved by native PAGE in 15% gels and imaged using a Typhoon Trio instrument. The positions of the ASO:RNA duplexes (“duplex”) and ssRNA are shown at right. (**A-C**) Each panel represents data from one of three reproducible independent experiments.

Numerous studies have shown that the incorporation of LNA residues strongly increases the binding of ASOs to their targets [20,59]. Consistent with this, the melting temperature of the LD4676:DNA duplex was 20°C higher than the Tm of the D4676:DNA duplex (Fig 5A). The effect of LNA residues on the Tm of the ASO:RNA duplex was even more prominent: the Tm increase was greater than 30°C at all analyzed target RNA concentrations (Fig 5B). Consistent with the results obtained for all-DNA ASOs (Fig 1B, 1C), the incorporation of 8-oxo-dG residues reduced the Tm of duplexes of LNA:DNA gapmer ASOs with both DNA (Fig 5A) and RNA (Fig 5B) targets. For both targets, the decrease in Tm (LDM4676 *versus* LD4676) was between 5 and 10°C (Fig 5A and 5B).

To investigate how the reduced Tm affects the efficiency of ASO:RNA duplex formation, a 37-nt ssRNA that contains the target site of siRNA 4676 (Fig 5C) was labeled with ^33^P and incubated with D4676, DM4676, LD4676 or LDM4676 (Table 1) at physiological temperature (37°C). ASO:RNA duplexes were detected immediately after the mixing of the ssRNA target and ASO (Fig 5C). The 8-oxo-dG residues in all-DNA ASOs clearly reduced the efficiency of duplex formation. This effect was likely due to the reduced Tm of DM4676. The LNA/DNA gapmer ASO formed duplexes at least as efficiently as non-modified all-DNA ASO. Interestingly, 8-oxo-dG residues did not inhibit LNA/DNA gapmer ASO:RNA duplex formation (Fig 5C), most likely because the Tm of the LDM4676:RNA duplex remained sufficiently high to ensure its effective formation.

### 8-oxo-dG residues have no adverse effects on RNase H-mediated cleavage of ASO:RNA duplexes

LD4676 and LDM4676 formed duplexes with target RNA with similar efficiencies (Fig 5C), and the duplexes formed by LD4676 were more stable (Fig 5B). Nevertheless, in a cell-based assay, LDM4676 was a somewhat more efficient inhibitor of HCV replication (Fig 4A). Therefore, we asked whether there were any differences in the ability of these duplexes to undergo RNase H-mediated target RNA cleavage. As human RNase H enzymes are not commercially available, we used bacterial RNase H, which has very similar fold and active center organization [60] and shares many enzymatic properties with human RNase H enzymes [61,62].

**Fig 6:**
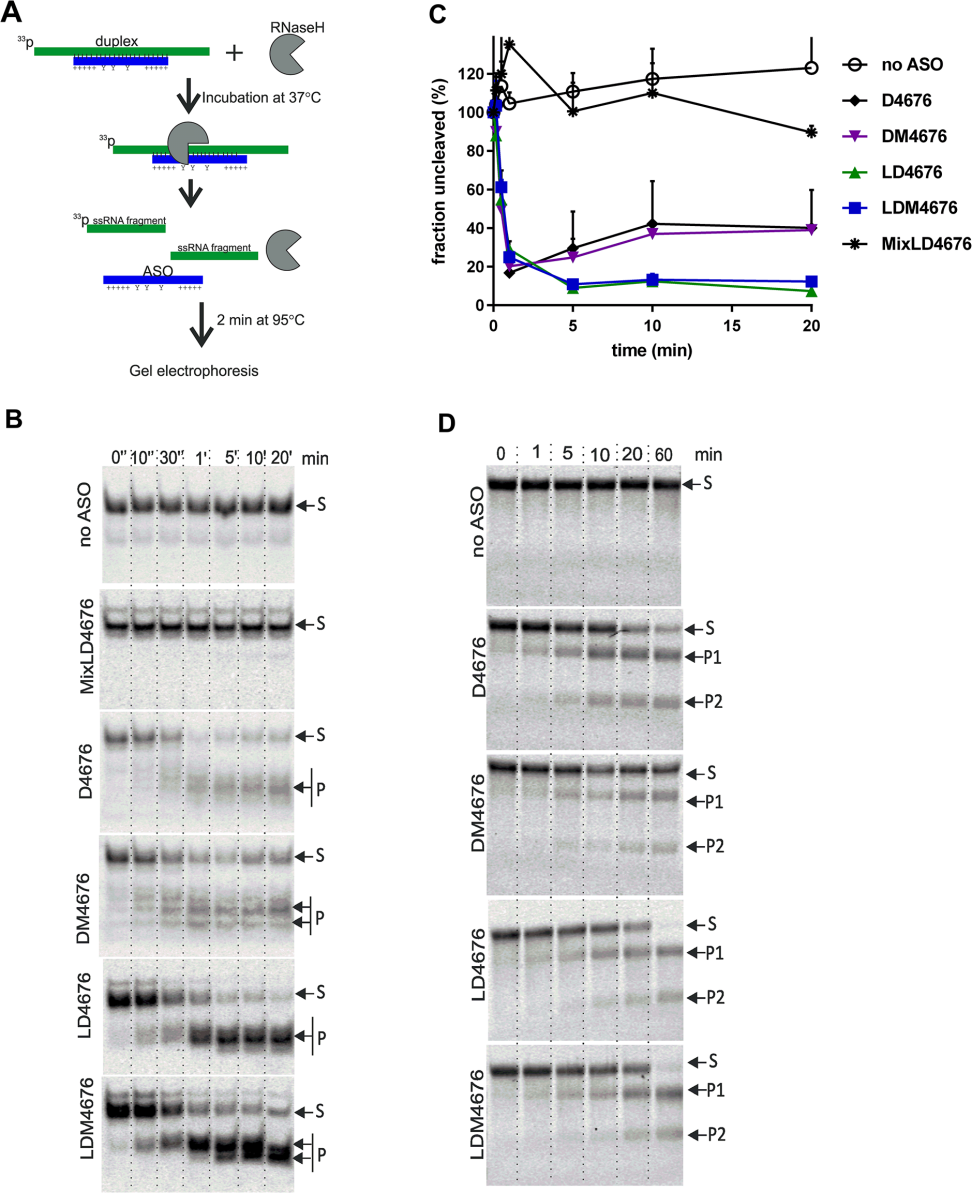
RNase H-mediated degradation of pre-formed ASO:RNA duplexes and *in vitro*-synthesized RNAs targeted by ASOs. **(A)** Schematic of the experimental setup for panels B and C. Applicable for some ASOs: Y, 8-oxo-dG residue; +, LNA sugar base. **(B, C)** Cleavage of pre-formed ASO:target RNA duplexes by RNase H. **(B)** Five femtomoles of ^33^P-labeled substrate was treated with RNase H for the indicated times. The reaction products were collected, denatured by heating at 95°C for 2 min and analyzed by PAGE in native 15% gels. Arrows at right point to the substrate (S) and major cleavage product(s) (P). Results from one of three independent reproducible experiments are shown. **(C)** Kinetics of RNase H cleavage of different ASO:RNA duplexes. The amounts of radioactivity remaining in the uncleaved substrate were quantified using a Typhoon Trio instrument. Quantifications were performed for each gel. The obtained values were normalized to the radioactivity present in the substrate before adding RNase H (set to 100%). Each point corresponds to the average of three independent experiments. Error bars indicate the standard deviation. **(D)** Cleavage of FR3131 RNA by RNase H in the presence of different ASOs. The RNA and ASOs were mixed and incubated at 37°C for 10 min; then, RNase H was added to the reaction mixture. RNA samples were collected at the indicated time points and analyzed by electrophoresis in native 0.8% TAE agarose gels. The results from one of three independent reproducible experiments are shown. S: substrate; P1 and P2: cleavage products.

Pre-formed ASO:RNA duplexes, consisting of D4676, DM4676, LD4676, LDM4676 or an LNA/DNA mixomer oligonucleotide designated MixLD4676 (Table 1), and ^33^P-labeled 37-nt target RNA molecules were used to estimate the kinetics of RNase H-mediated cleavage (Fig 6A). As expected, RNase H had no effect on ssRNA (Fig 6B and 6C). Similarly, due to the absence of the obligatory 6-bp DNA:RNA duplex stretch required for RNase H activation [19,62], RNase H could not cleave the RNA strand in the MixLD4676:RNA duplex (Fig 6B and 6C). In contrast, D4676:RNA and DM4676:RNA duplexes were rapidly cleaved. However, after 0.5 min, the reaction plateaued, leaving 20–30% of the substrate uncleaved. The cleavage of duplexes containing LD4676 or LDM4676 initially followed similar kinetics, but cleaved products continued to accumulate for 5 more min, reducing the levels of intact substrates to 10% of the initial amount (Fig 6B and 6C). As no significant differences in the cleavage of duplexes formed by D4676 and LD4676 and their respective modified ASOs were observed (Fig 6C), it was concluded that the incorporation of 8-oxo-dG into the ASO had no effect on the overall efficiency of RNase H-mediated cleavage of pre-formed ASO:RNA duplexes.

### 8-oxo-dG modifications alter the specificity of ASO-mediated RNase H cleavage of target RNA

Upon closer examination of the RNase H cleavage assay results, we noticed that the pattern of cleavage products generated by ASOs with and without 8-oxo-dG modification dramatically differed. In particular, a single major cleavage product was clearly dominant for the LD4676:RNA duplex, whereas for the LDM4676:RNA duplex, at least two major cleavage products of roughly the same abundance were observed (Fig 6B). A similar effect, although less pronounced, was observed for duplexes containing the all-DNA oligonucleotides D4676 and DM4676. Thus, the presence of 8-oxo-dG residues in ASOs triggered multiple cleavages by RNase H in the targeted DNA:RNA duplex region.

### Efficiency of RNase H-mediated cleavage of target RNA molecules correlates with the efficiency of ASO:RNA duplex formation

Next, we studied the effect of 8-oxo-dG residues on ASO:target RNA duplex formation and subsequent cleavage by RNase H in a single reaction. To account for the possible influence of RNA secondary structure, full-length HCV replicon RNA was used as a target. However, this RNA underwent slow degradation in the absence of ASOs. Therefore, FR3131, an *in vitro*-synthesized 3131-nt fragment of HCV replicon RNA, was used as a target. This target RNA was pre-incubated with D4676, DM4676, LD4676 or LDM4676 for 10 min at 37°C; next, RNase H was added to the reaction mixture. In the absence of ASO, FR3131 RNA remained stable (Fig 6D). In the presence of D4676, the targeted RNA was cleaved into two fragments of the expected sizes (Fig 6D). 8-oxo-dG residues clearly reduced the cleavage efficiency in the presence of all-DNA ASOs (Fig 6D). As the modification had a similar effect on ASO:RNA duplex formation (Fig 5C) but did not affect the degradation of pre-formed ASO:RNA duplexes (Fig 6B and 6C), it was concluded that the rate-limiting step for RNase H-mediated target RNA cleavage was ASO:RNA duplex formation. In the presence of LNA/DNA gapmer ASOs, nearly complete cleavage of FR3131 RNA occurred (Fig 6D). This result is consistent with the more complete degradation of pre-formed LNA/DNA gapmer ASO:RNA duplexes (Fig 6C). In the context of LNA/DNA gapmers, 8-oxo-dG residues had little, if any, effect on RNase H-mediated cleavage of target RNA (Fig 6D). Due to the large sizes of the FR3131 cleavage products (approximately 1250- and 1850-nt), the effect of 8-oxo-dG residues on the precise cleavage positions within the ASO:RNA duplex could not be observed in this experiment.

### Incorporation of 8-oxo-dG residues increases the stability of oligonucleotides in human serum

**Fig 7:**
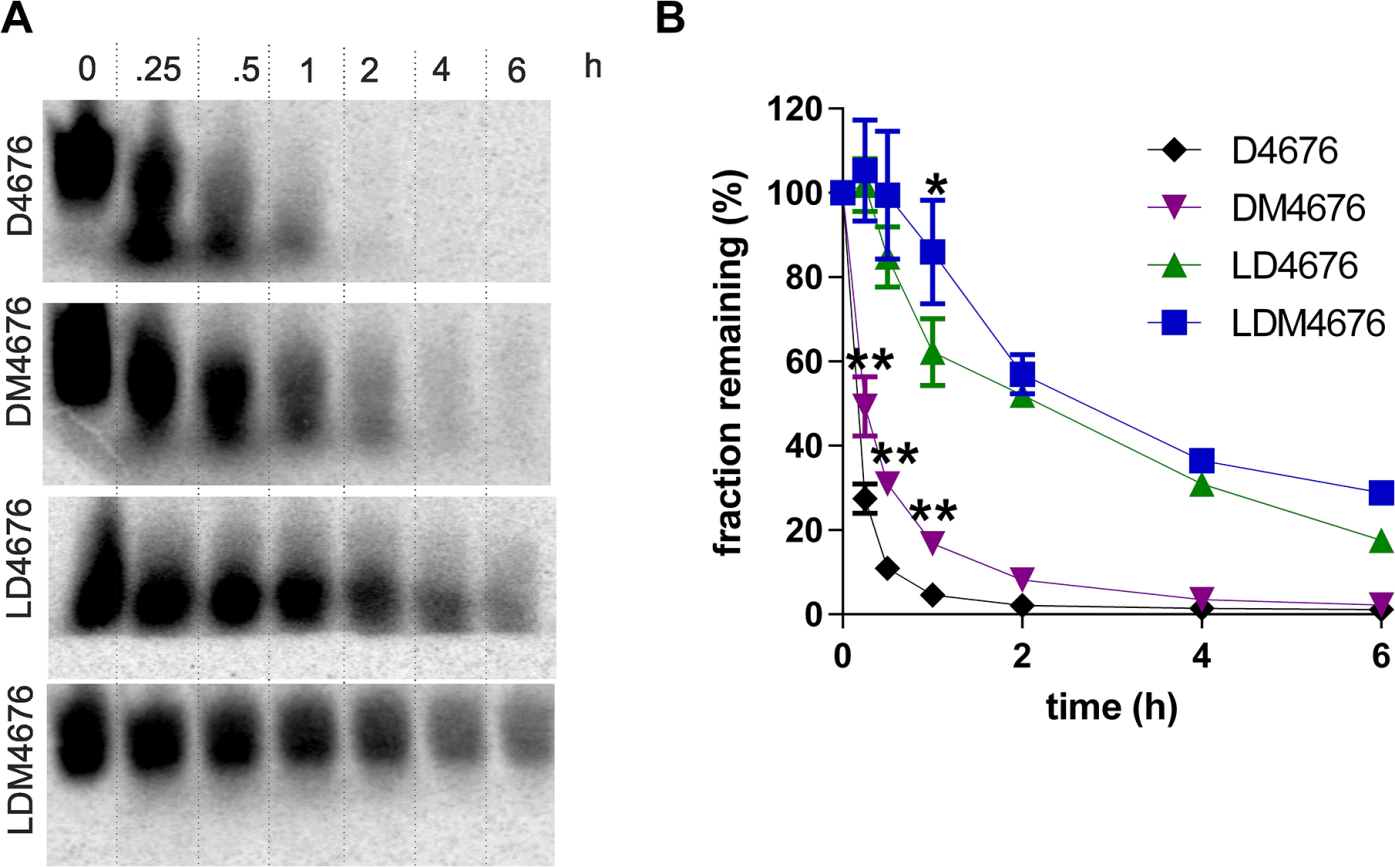
8-oxo-dG residues increase the stability of ASOs in human serum. **(A)** ^33^P-labeled D4676, DM4676, LD4676 and LDM4676 oligonucleotides (Table 1) were incubated in human serum at 37°C. Aliquots were collected at the indicated time points and analyzed by PAGE in native 15% gels. The results from one of three independent reproducible experiments are shown. **(B)** Quantitative representation of the stability of the oligonucleotides. The amounts of radioactivity remaining in the uncleaved ASOs were quantified using a Typhoon Trio instrument. Quantifications were performed for each gel. The obtained values were normalized to the radioactivity present in the substrate before incubation in human serum (set to 100%). The fraction of remaining ASO is shown as a single exponential decay function. Each point corresponds to the average from three independent experiments. Error bars indicate the standard deviation; * p<0.05 and ** p<0.01 (Student’s t-test).

Another factor that affects the potency of ASOs is their stability in biological environments. To determine whether 8-oxo-dG residues affected the stability of all-DNA and LNA/RNA gapmer ASOs, ^33^P-labeled D4676, DM4676, LD4676 and LDM4676 were incubated in human serum for 0, 0.25, 0.5, 1, 2, 4 and 6 h. Consistent with previous data [19], the LNA/DNA gapmer oligonucleotides were much more stable than their all-DNA counterparts (Fig 7A and 7B). The half-life of D4676 was less than 8 min, whereas the half-life of LD4676 was more than 10 times longer. Importantly, the incorporation of 8-oxo-dG residues significantly increased the stability of both types of oligonucleotides (Fig 7B). The half-life of DM4676 (15 min) was nearly twice of that of D4676. Similarly, 8-oxo-dG modification increased the half-life of the LNA/DNA gapmer oligonucleotide from 90 min to 130 min. The mechanism(s) responsible for this stabilization are outside of the scope of current study and remain unknown.

## Discussion

The difficulties hampering the clinical use of ASO drugs include their low efficiency, low bioavailability, rapid degradation and unwanted side effects (typically off-target effects). Thus, synthetic ASOs often contain one or more modifications aimed at improving the properties of the compound. The common theme in ASO design is to achieve increased ASO:target duplex stability [5], which is typically associated with higher ASO potency [63]. However, ASOs with a very high Tm also tend to bind to secondary targets, and their ability to trigger RNase H-mediated target RNA degradation may be reduced or even completely abolished [25]. Consequently, such ASOs are generally highly efficient only if they target short microRNAs or if their binding site overlaps with the region directly involved in translation initiation. This limitation drastically reduces the number of suitable ASO binding sites in mRNA molecules. Here, we provide a novel platform for ASO target site selection and show that the destabilization of the ASO:target-RNA duplex by the introduction of 8-oxo-dG modifications has several advantages over conventional LNA/DNA gapmer ASO design.

The initial idea of using 8-oxo-dG residues was based on theoretical quantum calculations. It was hypothesized that the antisense activity of oligonucleotides containing 8-oxo-dG may be increased due to the ability of corresponding nucleobases to acquire tautomeric forms, which enables enhanced binding to complementary normal nucleobases [33]. However, experimental data revealed that the incorporation of 8-oxo-dG residues reduced the Tm of ASO:DNA and ASO:RNA duplexes (Figs 1, 5A and 5B), probably because the major tautomer of 8-oxo-dG in aqueous solution is the 6,8-diketo form [64], which binds to complementary dC residues more weakly than does the standard dG residue [29]. Thus, the contribution of the strongly binding minor zwitterionic tautomer of 8-oxo-dG (Fig 1A) may be too small to be detected. The overall oligonucleotide Tm analysis, however, did not exclude the possibility that different tautomeric forms of 8-oxo-dG might play an important role in base pairing.

The efficiency of ASOs critically depends on the physical accessibility of its target site. Very limited information is available about the actual topological structures of mRNA in cells, where they form RNA-protein complexes. In this study, the attempts to target sequences with specific nucleotide compositions or regions lacking predicted secondary structures (calculated using the Minimal Free-Energy method) were unsuccessful. High-throughput selective 2′-hydroxyl acylation analyzed by primer extension (SHAPE) has been used to resolve the secondary structure of the HIV-1 RNA genome [65]. However, for HCV, only the structures of short non-coding terminal fragments of the genome have been analyzed using SHAPE [66–68]. Furthermore, some viral RNAs exist in different conformations, and corresponding changes are integral to the regulation of the viral infection cycle [69,70]. Hence, there is the need for empirical screening of targeted RNA.

The low efficiency of all-DNA ASOs (Fig 4A) hinders their use as screening tools. Therefore, we reasoned that siRNAs, which can be highly efficient and are relatively inexpensive, might represent suitable tools for performing such screening. This approach led to the identification of two potent siRNAs that were 3-fold more efficient than positive control siRNAs targeting the non-structured reporter part of the replicon RNA construct (Fig 2). Therefore, we hypothesized that the ability of these siRNAs to suppress HCV replication would serve as a good indication that the corresponding regions of the HCV RNA genome are accessible not only to the cellular RNA silencing machinery but also to ASOs. An additional benefit of this approach is that it allows the direct comparison of the effects of ASOs and siRNAs targeting the same sequences. It would be interesting to compare the siRNA mapping data with the SHAPE-based structures of corresponding RNAs. If there is a correlation between the observed efficiencies of siRNAs and the determined high-order structures of mRNAs (or a viral RNA genomes), siRNAs might become useful tools for probing the high-order structure of RNAs inside the cell.

Experiments with an *in vitro-*synthesized fragment of HCV RNA (Fig 6D) and with the HCV replicon cell line (Fig 3) confirmed that modified LNA/DNA gapmer oligonucleotides use an antisense mode of action. Although the insertion of three 8-oxo-dG residues into the LNA/DNA gapmer ASO did not reduce the EC_50_, it somewhat increased the inhibition of virus replication at concentrations exceeding the EC_50_ (Fig 4A). In part, this effect can be attributed to the increased stability of modified compounds in a biologically relevant environment (Fig 7).

Modified ASOs, where LNA residues are dispersed over the length of the compound, were found to lack an antiviral effect. Consistent with previous studies [71], MixLD4676 and other similarly designed ASOs were unable to trigger RNase H-mediated degradation of the RNA strand in ASO:RNA duplexes (Fig 6B and 6C). These data, similar to those published by Laxton and co-workers [72], highlighted the importance of RNase H-mediated cleavage for the anti-HCV activity of ASOs. RNase H-mediated RNA degradation also depends on the ability of an ASO to form a duplex with its target. Using a short target RNA molecule, duplex formation was shown to be fast, and, as expected, its efficiency correlated with the ASO Tm (Fig 5). Somewhat unexpectedly, the initial speed of degradation of pre-formed ASO:RNA duplexes depended little, if at all, on the modifications introduced into the ASO (Fig 6C). Instead, there was a clear correlation between the efficiency of ASO:RNA duplex formation (Fig 5C) and the efficiency of RNase H-mediated cleavage of FR3131 RNA (Fig 6D), suggesting that in the *in vitro* RNA cleavage experiment, the efficiency and speed of ASO:RNA duplex formation was the rate-limiting step. However, due to different conditions, including differences in the specificity and abundance of RNase H enzymes in living human cells, the possibility cannot be excluded that the *in vivo* activity of ASOs does not necessarily correlate with its binding to small model substrates. Indeed, human cells have two different RNase H enzymes. Although the human RNase H1 shares many enzymatic properties with the bacterial enzyme, there are differences. Human RNase H1 binds to A-type RNA:DNA duplexes with much greater activity than bacterial RNase H and displays a strong positional preference for cleavage, i.e., it cleaves between 8 and 12 nucleotides from the 5′-RNA-3′-DNA terminus of the duplex [62]. Therefore, it would be interesting to study whether the presence of 8-oxo-dG residues affects the cleavage specificity of human RNase H enzymes. If this indeed is the case, then such modifications might be particularly useful for constructing ASOs that target viruses that rapidly develop resistance to siRNAs [73,74] or ASOs against RNAs with pre-existing variations in the target site.

Although LDM4676 displayed high activity in different *in vitro* assays, its value as a potential HCV inhibitor critically depends on its *in vivo* performance. However, such studies are hampered by the lack of low-cost small-animal models and by the high costs of ASOs containing LNA bases, 8-oxo-dG residues and phosphorothioate modifications (needed for increased *in vivo* stability) in its backbone. However, should such types of compounds be highly active *in vivo*, they could contribute to the development of ASO-based HCV treatments. Miravirsen, the first experimental drug of this type, has already been successfully used in clinical trials [26,75,76]. However, miravirsen targets an important cofactor of HCV genome expression and replication, whereas the LDM4676-type ASO targets the HCV genome itself. Combinations of drugs with different mechanisms of action have been key for the successful treatment of chronic infections caused by viruses capable of rapidly developing drug resistance. It is also likely that despite recent progress in the development of orally deliverable oligonucleotide drugs [77,78], a subcutaneous injection will remain the main delivery method for ASOs or their combinations. Therefore, the long half-life of ASOs, allowing once-a-week administration [26,79], represents an important property of such compounds. Correspondingly, increases in the serum half-life, resulting from the insertion of 8-oxo-dG residues (Fig 7), may represent another important benefit of modified ASO compounds.

In this study, multiple obstacles that are commonly encountered in the development of new and efficient ASOs were addressed using unconventional and efficient approaches. For the first time, several highly accessible ASO target sequences in the heavily structured coding region of the RNA genome of HCV were revealed. RNAi-based screening represents an efficient and reliable general method for ASO target site selection. This study also provides an important set of findings concerning the use of naturally occurring, minimally modified nucleobases in ASOs. In contrast to nucleobases that contain bulky modifications, the 8-oxo-dG residues reduced the Tm of ASO:RNA duplexes and had no negative effects on RNase H-mediated degradation of RNA strands in ASO:RNA duplexes. Instead, 8-oxo-dG residues facilitated cleavage by RNase H at multiple positions within the target region. Furthermore, the incorporation of 8-oxo-dG residues increased the stability of ASOs in human serum. These effects, possibly combined with other properties of modified nucleobases (such as strong base-pairing of their minor tautomeric forms), outweigh the negative effects on the overall Tm of the ASO. This enabled us to obtain modified LNA/DNA gapmer oligonucleotides with EC_50_ values similar to their non-modified counterparts but capable of almost completely inhibiting HCV replication in replicon cell lines at higher concentrations.
